# Surface Characteristics and Bioactivity of a Novel Natural HA/Zircon Nanocomposite Coated on Dental Implants

**DOI:** 10.1155/2014/410627

**Published:** 2014-04-16

**Authors:** Ebrahim Karamian, Amirsalar Khandan, Mahmood Reza Kalantar Motamedi, Hesam Mirmohammadi

**Affiliations:** ^1^Department of Materials Engineering, Najafabad Branch, Islamic Azad University, Isfahan, Iran; ^2^Young Researchers and Elite Club, Khomeinishahr Branch, Islamic Azad University, Isfahan, Iran; ^3^Dental Students Research Center, School of Dentistry, Isfahan University of Medical Sciences, Isfahan, Iran; ^4^Dental Materials Research Center, Department of Restorative Dentistry, Isfahan University of Medical Sciences, Isfahan, Iran; ^5^Department of Cariology Endodontology Pedodontology, Academic Centre for Dentistry Amsterdam (ACTA), Universiteit van Amsterdam and Vrije Universiteit, Amsterdam 1081 JD, The Netherlands

## Abstract

The surface characteristics of implant which influence the speed and strength of osseointegration include surface chemistry, crystal structure and crystallinity, roughness, strain hardening, and presence of impurities. The aim of this study was to evaluate the bioactivity and roughness of a novel natural hydroxyapatite/zircon (NHA/zircon) nanobiocomposite, coated on 316L stainless steel (SS) soaked in simulated body fluid (SBF). NHA/zircon nanobiocomposite was fabricated with 0 wt.%, 5 wt.%, 10 wt.%, and 15 wt.% of zircon in NHA using ball mill for 20 minutes. The composite mixture was coated on 316L SS using plasma spray method. The results are estimated using the scanning electron microscopy (SEM) observation to evaluate surface morphology, X-ray diffraction (XRD) to analyze phase composition, and transmission electron microscopy (TEM) technique to evaluate the shape and size of prepared NHA. Surfaces roughness tester was performed to characterize the coated nanocomposite samples. The maximum average *R*_*a*_ (14.54 **μ**m) was found in the NHA 10 wt.% of zircon coating. In addition, crystallinity (*X*_*c*_) was measured by XRD data, which indicated the minimum value (*X*_*c*_ = 41.1%) for the sample containing 10 wt.% of zircon. Maximum bioactivity occurred in the sample containing 10 wt.% of zircon, which was due to two reasons: first, the maximum roughness and, second, the minimum crystallinity of nanobiocomposite coating.

## 1. Introduction

Dental implants are well-accepted and predictable treatment modalities for rehabilitation of patients with partial and complete edentulism. Dental implantation has become an established treatment method since its appearance for over 40 years. The future would probably see bioactive surfaces and additives that stimulate the bone growth. According to Albrektsson et al. several factors can affect the osseointegration, including implant material, implant design, implant surface characteristics, status of the bone, surgical technique, and implant loading conditions [1]. Nowadays, there is an effort to speed up the osseointegration by improving the implant-to-bone interface chemically (by incorporating inorganic phases on or into the titanium oxide layer) or physically (by increasing the level of roughness) [2]. There are some advantages regarding surface modified implants, including (a) providing a better stability between bone and implant during healing process, established by a greater contact area, (b) providing a surface configuration that may retain the blood clot, and (c) stimulating the bone formation and healing process [3]. There are several methods to improve the quality of implant surface; one of them is adding bioactive materials to the surface of the dental implant to induce osteoconductivity.

Hydroxyapatite (HA), [Ca_10_(PO_4_)_6_(OH)_2_], is a calcium phosphate bioceramic material, with osteoconductivity and excellent biocompatibility. However, apatite layers coating the surface of the implant substrates improve the bone response. For this reason, it has been used successfully in dentistry for many years. HA products are well known as implantable ceramics for hard tissue reconstitution. An example of such application is a coating applied onto dental implants, providing enhanced fixation of the implant for the human bone [4]. HA-coated implants have often been used in load-bearing applications, because HA directly bonds to the bone and promotes the new bone formation, necessary for the implant osseointegration [5–8]. Despite advantages of HA coatings, it has some disadvantages. Enhanced susceptibility to bacterial plaque colonization and HA coating integrity are two major concerns related to HA-coated implants [9]. Such bacterial accumulation can start a chain reaction leading to lesions, and then other risk factors may combine to worsen the condition [10]. An exposed rough surface can lead to increased bacterial plaque accumulation and eventually peri-implantitis [11]. Therefore, the susceptibility of peri-implantitis should be taken into account when using HA-coated implants. It is known that the HA surface degrades and in some instances separates; hence, it has been a trend to substitute the coatings with roughened surface dental implants, which also incidentally presents better and faster integration. Although HA can be a good choice to be applied on the surface of the dental implants as a bioactive and biocompatible material, its mechanical properties (mainly the strength, roughness, and fracture toughness) need to be improved for application in load-bearing parts, since they have limited its usage to nonload-bearing parts due to their poorness [12–16]. This goal can be achievable by adding some materials to the HA coating, which has already been done to improve its final coating characteristics. These additives aim to enhance various properties of the coating, including bioactivity [17], thermal stability, and its mechanical properties.

Zircon is a tetragonal mineral, consisting of a silicate of zirconium. The group of silicate biomaterials has the ability to release silicate ions at a definite concentration which helps the osteoblasts to grow and differentiate [18], leading to bone formation. Therefore, this characteristic of zircon could be beneficial for the surface usage of dental implants. Considering several studies evaluating the additives in the dental implant coatings (such as zirconia (ZrO_2_), ZrO_2_-Al_2_O_3_, etc.), we sought to find a new additive with better mechanical and biological characteristics. Therefore, the bioactivity and roughness of zircon (ZrSiO_4_) added to the HA coating in simulated body fluid (SBF) were evaluated in this study. SBF is an inorganic solution with a similar composition to human blood plasma without organic components. Several studies performed on nanobioceramic coatings include monitoring the behavior of a material when submersed in the saline solution [19] or SBF [20].

Plasma spraying technique is the most commonly used method for the HA coatings application. This is a thermal spraying process in which powder particles are melted in a high temperature plasma flame and propelled towards a substrate material to form a coating. The advantages of this process include high coating adhesion strength and also high deposition rate, which allow rapid formation of the coatings [21]. Surface roughness can be described using a number of different parameters, while *R*_*a*_ (absolute value) is by far the most common. Other common parameters include *R*_*z*_ and *R*_max⁡_.

The aim of this study was to evaluate the bioactivity and roughness of novel natural HA/zircon (NHA/zircon) nanobiocomposite coating, soaked in the SBF solution.

## 2. Materials and Methods

### 2.1. NHA Extraction

The present research was an experimental study. Bovine bones were boiled for 12 h to remove the attached flesh and fat. Afterwards, bones were dried at 110°C for 2 h to shed the moisture. To prevent blackening with soot during heating, bones were cut into small pieces with 10 mm thickness and heated at 500°C (bone ash) for 2 h in air to allow evaporation of organic substances. The resulting black bone ash was heated for 3 h at 850°C. This synthesis is called thermal decomposition of bone resource to create NHA. The elemental chemical analysis of NHA using X-ray fluorescence (XRF) (Philips PW1606) is shown in Table 1.

### 2.2. Fabrication of NHA on the Implant Cores

In this work, 316L stainless steel (SS) was used as substrate. The elemental analysis and the elemental composition (wt.%) of SS were C 0.03, Si 0.8, Mn 1.3, Cr 17.55, Ni 13, Mo 3.1, *P* ≤ 0.04, *S* ≤ 0.03, and Fe as the balance. Specimens with dimensions 20 × 10 mm (diameter × thickness) were cut with CNC Wire Cut EDM Machine (DK7732F-china suppliers). The test was performed on cylindrical porous and all the specimens were immersed in Analar grade H_2_SO_4_ (specific gravity = 1.84) for 1 h in different volume concentrations varying from 5% to 25% at ambient temperatures. The samples were polished with 100–1200 grit silicon carbide (SiC) paper. In order to produce a scratch-free, mirror-finish surface, final polishing was performed using 4,000 grit silicon carbide papers. The polished specimens were investigated by the optical microscope to ensure the absence of pits or scratches on the surface. Specimens were cleaned with acetone and thoroughly washed with distilled water. Afterward, specimens were surface treated by grit blasting in order to obtain a desired roughness of surface for better adhesion of coating to the substrate. After the surface treatment process, the specimens were cleaned with distilled water and ultrasonic device as a cleaner technique. NHA/zircon nanobiocomposite was fabricated with 0 (control), 5, 10, and 15 wt.% of zircon in NHA and coated on the surface of the 316L SS cores using plasma spray technique.

### 2.3. Phase and Composition Analysis

Phase structure analysis was performed by X-ray diffraction (XRD) (Philips X'Pert-MPD diffractometer with Cu K*α* radiation (*λ*_1_ = 0.15418 nm) over the 2*θ* range of 20–80). The obtained experimental patterns were compared to the standards compiled by the Joint Committee on Powder Diffraction and Standards (JCDPS) which involved card number 09-432 for HA. The crystallite size of prepared powders was determined using XRD patterns and modified Scherrer equation. Scanning electron microscopy (SEM) analysis evaluations were performed using a Philips XL30 (Eindhoven, The Netherlands) to investigate the morphology. The Ca/P ratio was determined using energy-dispersive X-ray spectroscopy (EDX) microanalysis (FEI Quanta 200 ESEM equipped with an EDX EDS device). Samples were coated with Au using spraying, high vacuum, and 25 kV accelerating voltage. Ca and P ions contents were measured from four spots (Figure 3), and consequently the average was calculated. Transmission electron microscopy (TEM) technique (Philips CM 200 FEG: Eindhoven, The Netherlands) was utilized to evaluate the shape and size of prepared HA.

### 2.4. Surface Roughness Evaluation

The roughness (i.e., *R*_*a*_) of each sample was measured in three directions. Tour measurements were taken for each sample and then their average was determined.

### 2.5. *In Vitro* Bioactivity Evaluation

*In vitro* bioactivity was investigated by soaking the samples in SBF solution. The SBF solution was prepared according to the procedure described by Kokubo and Takadama [20]. Ion concentrations of SBF are similar to those in human blood plasma. Coated samples were soaked in the cell SBF solution (pH 7.4) at 37°C for 1, 7, and 14 days at a solid/liquid ratio of 1 mg/mL, without refreshing the soaking medium. Element analysis of SBF and physiological saline are shown in Table 2. After the predicted soaking time finished, the disc samples were rinsed with deionized water and dried in an oven at 110°C for 1 h. Samples weight loss was calculated by the following equation:

(1) $$\begin{array}{c} \text{Weight}\;\;\text{Loss}\;\;\text{percentage}\;\;\left(\text{WL}.\%\right)=\frac{W-W_{0}}{W_{0}}\times 100, \end{array}$$

where *W* and *W*_0_ are primary and secondary weights, respectively.

## 3. Results

### 3.1. Phase and Composition Analysis of the XRD Results

**Figure 1:**
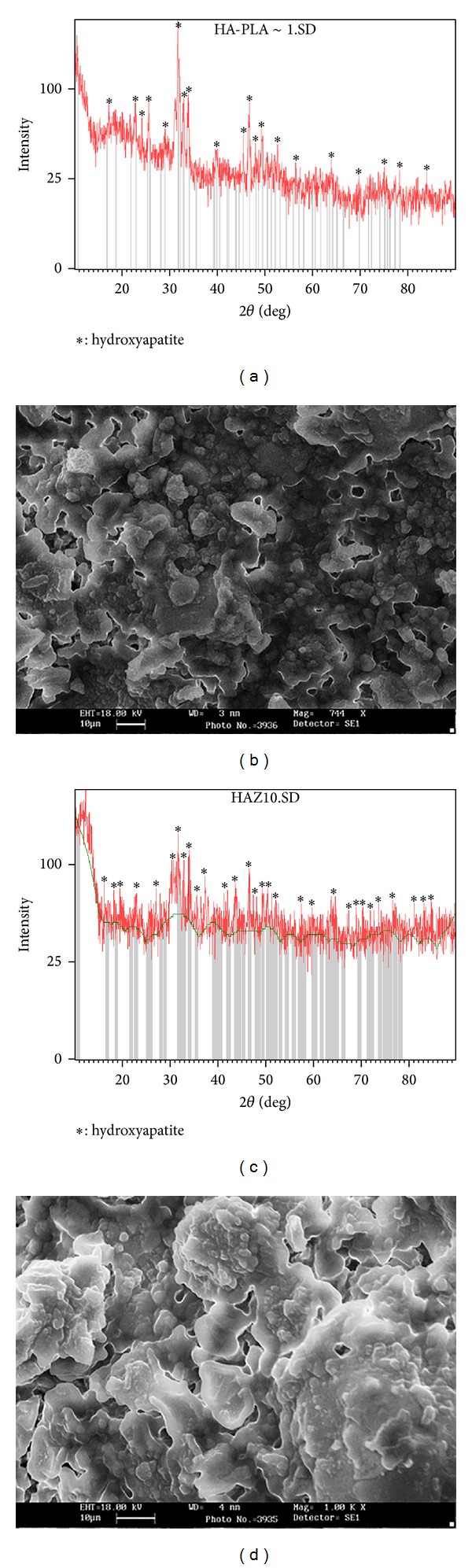
(a) XRD pattern and (b) SEM micrograph of the NHA 0% coated by plasma spraying. (c) XRD pattern and (d) SEM micrograph of the NHA 10% zircon coated by plasma spraying.

Figure 1 shows the XRD patterns and SEM images of the NHA 0% and 10% zircon coatings.  Figure 1(a) indicates that the only existing phase is for HA (all the peaks belong to HA). However, the peaks observed in the XRD pattern of NHA 10% zircon had a decrease in intensity and an increase in width compared to NHA 0% zircon (Figures 1(a) and 1(c)). In addition, the XRD peaks corresponding to NHA 10% zircon shifted slightly in comparison with NHA 0% zircon (Figures 1(a) and 1(c)). The crystallite sizes of the prepared NHA samples with different degrees of zircon content calculated using XRD data are shown in Table 5. The crystallite size of the obtained nanopowders is in the range of 25.5–32 nm. Determination of crystallite sizes from XRD peak widths makes assumptions on crystallite shape and crystallite size.

### 3.2. Scherrer Equation

**Figure 2:**
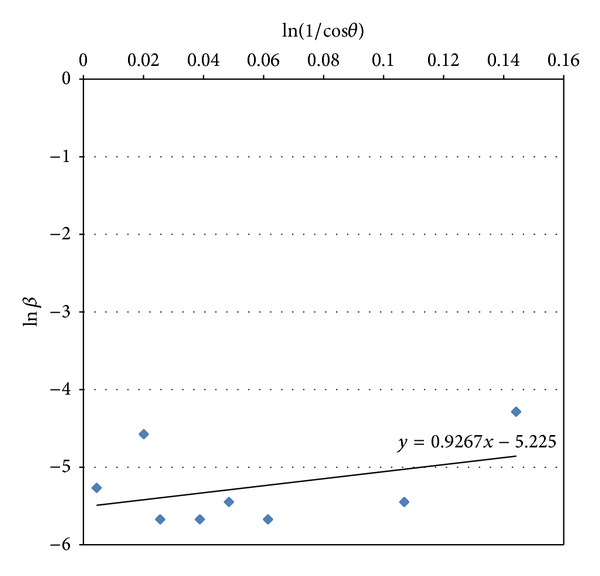
Plot of ln*β* versus ln(1/cos *θ*) of the HA sample heated at 850°C for 3 h.

The modified Scherrer equation is advantageous for decreasing the sum of absolute values of errors, ∑(±Δln⁡*β*)^2^, and producing a single line through the points to give a single value of intercept ln⁡(*kλ*/*L*) [22]. At this sample, shown in Figure 2, the linear regression plot is obtained as *y* = 0.9267*x* − 5.225. This is equivalent to ln⁡*β* = ln⁡(1/cos⁡*θ*) + ln⁡(*kλ*/*L*). From this line, the intercept is −5.225, *e* ^−5.225^ = *kλ*/*L*, and *L* = 25.5 nm. Thus, HA crystallite size was 25.5 nm.

### 3.3. SEM, EDX, and TEM Microstructures

**Figure 3:**
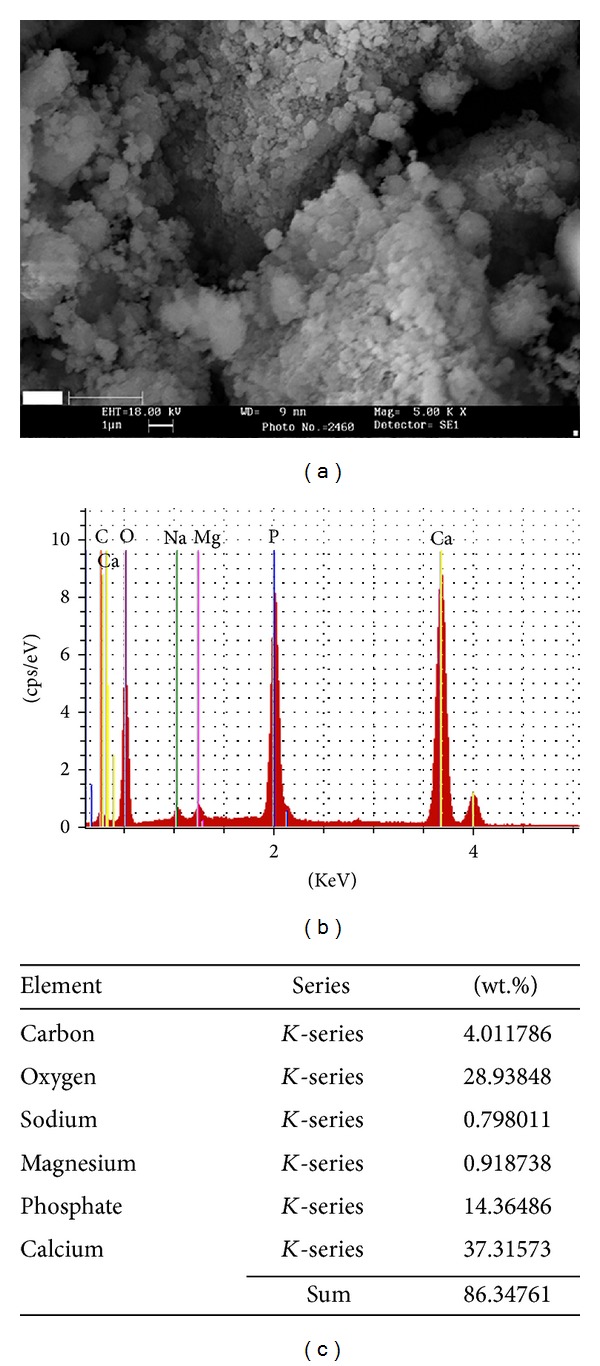
(a) SEM micrographs of the NHA powder, (b) EDX spectrum of the NHA powder, and (c) microchemical composition of the NHA powder determined by EDX.

Figure 3 shows the SEM micrographs of NHA powder. In all samples, we found nanoparticles of HA crystals, agglomerated with different dimensions. Morphology of particles is sphere and semisphere. Microchemical composition of the NHA sample was determined using EDX (Figure 3).

**Figure 4:**
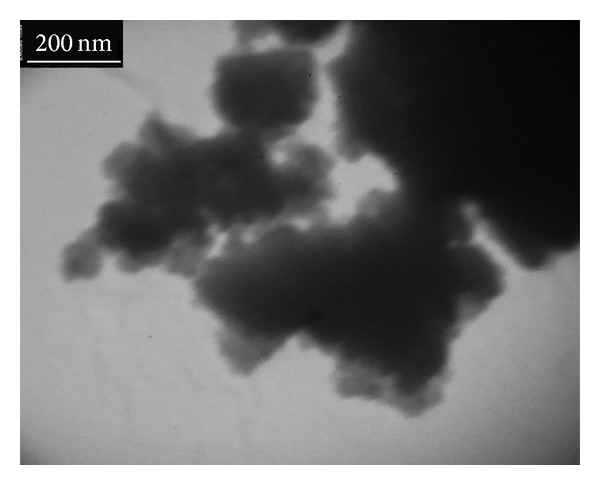
TEM micrograph of prepared NHA at 850°C for 3 h.

TEM technique was utilized to evaluate the shape and size of prepared NHA (Figure 4). The TEM image reveals that the powders are formed by agglomerates of irregular particles in 200 nm, consistent with values calculated from XRD data.

As shown in Figure 1, NHA crystal sizes are in micron size, some particles are spherical, and the others are in sharp shape. The agglomerated particles are composed of very fine particles.

### 3.4. Surfaces Roughness Results

The results are given in Table 3. Each sample was measured in three directions.Tour measurements were taken for each sample and then their average was determined. *R*_*a*_ was found to vary between 12.63 and 14.85 *μ*m. Coatings with four degrees of average roughness (*R*_*a*_: 12.63, 14.30, 14.54, and 12.85 *μ*m for NHA 0%, 5%, 10%, and 15% of zircon, resp.) were created. The maximum average *R*_*a*_ was found in the NHA 10% zircon sample.

### 3.5. *In Vitro* Bioactivity Evaluation

**Figure 5:**
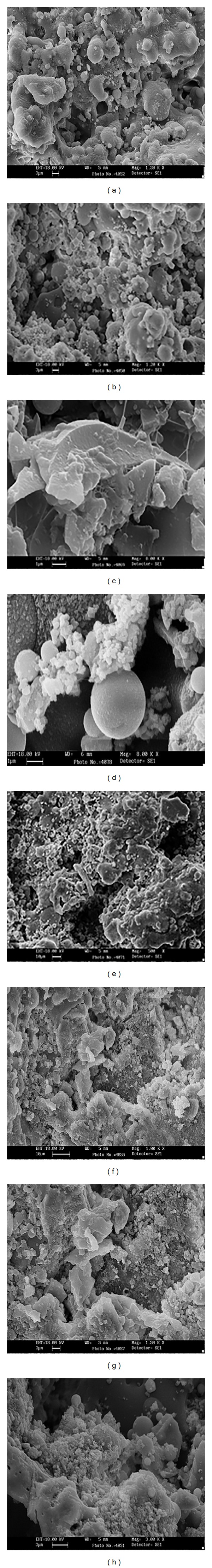
SEM observations of the coatings soaked in SBF for (a), (b) 1 week, NHA 10% zircon, (c), (d) 2 weeks, NHA 10% zircon, (e), (f) 1 week, NHA 0% zircon, and (g), (h) 2 weeks, NHA 0% zircon.

**Figure 6:**
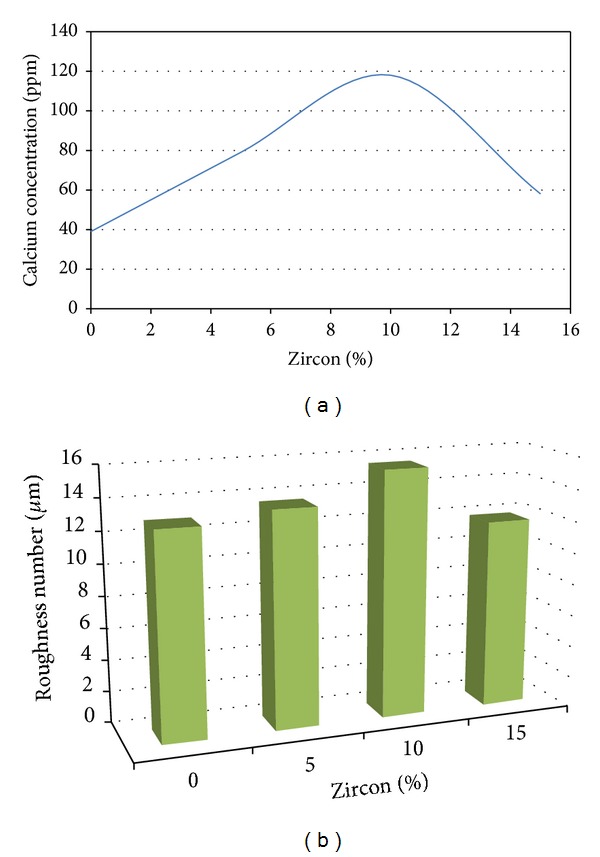
(a) Calcium concentration (Ca^2+^ released) after 2 weeks, coatings in SBF solution. (b) Average amount of roughness of the samples.

NHA/ZrSiO_4_ nanobiocomposite powder with different ZrSiO_4_ contents was prepared and coated by plasma spray method.* In vitro* bioactivity of nanopowders was investigated by soaking the powders in the SBF solution. SEM observations of the NHA 0% and 10% zircon coatings soaked in SBF for 1 and 2 weeks are illustrated in Figure 5. Results indicated that during 1, 7, and 14 days of soaking, shown in Table 4, calcium ions were released in the solution. As shown in Figure 6(a), there was a clear increase in calcium releasing in the sample containing 10% zircon. It depends on two reasons: (1) increase of the roughness (Figure 6(b)) and (2) decrease of the crystallinity of sample containing 10% wt. ZrSiO_4_ (*X*_*c*_ = 41.1%) (Table 5).

## 4. Discussion

In the present study, bioactivity of NHA/zircon coatings with various amounts of zircon (0 wt.%, 5 wt.%, 10 wt.%, and 15 wt.%) within SBF solution was evaluated using XRD and SEM interpretations. The maximum average *R*_*a*_ (14.54 *μ*m) was found in the NHA 10 wt.% of zircon coating. In addition, crystallinity (*X*_*c*_) was measured by XRD data, which indicated the minimum value (*X*_*c*_ = 41.1%) for the sample containing 10 wt.% of zircon. Maximum bioactivity occurred in the sample containing 10 wt.% of zircon.

According to Kokubo et al. [23–25],* in vitro* immersion of bioactive materials in SBF is thought to make* in vivo* surface structure changes in materials such as bioactive glass/ceramic. Therefore, to evaluate this, we soaked NHA/zircon samples in SBF solution. The grown layer is sometimes called a bone-like apatite because of the XRD pattern similar to that of bone apatite with broad peaks at 2*θ* angles of HA, which indicates a superfine grain of apatite crystallite [26]. Overall conclusion is that zircon is a very complex material. It is totally different from titanium, a metal used in dentistry in its pure (or commercially pure) form. We reached interesting findings from the present work that NHA/zircon (a) proved to be bioactive and (b) had high roughness and interesting microstructural properties.

There is a consensus regarding HA to be osteoconductive [27, 28] and biocompatible [29, 30]. The biocompatibility [31, 32], osteoconductivity, and osseointegration characteristics of zirconia are reported in several studies [33]. In addition, zirconia has a high flexural strength and fracture toughness. Accordingly, the combination of these characteristics could be found in the prepared coating, the novel NHA/zircon. In fact, this coating due to its bioactivity might speed up the osseointegration. However, this claim should be proved by future cell culturing and animal studies.

It is reported that topography and surface roughness positively affect healing process [34–36]. Dohan Ehrenfest et al. reported that possible physical improvement of the interface between bone and implant by increasing the level of roughness, leading to speed up osseointegration and its quality [2]. Human and animal studies have shown significantly greater bone and implant contact at rough surface implants compared to machined surface implants [37]. In 2009, a consensus report reached a conclusion that “moderately rough and rough surfaces provided enhanced bone integration compared with smooth and minimally rough surfaces” [38]. In this regard, as zircon increased the roughness of HA coating, the positive role of NHA/zircon coating for a fast and better healing might be supported. On the other hand, classic HA-coated implants were reported to have a higher occurrence of complications [39]. In fact, the surface roughness may have a significant impact on the contamination [2], amount and quality of plaque formation [40]. Especially in patients with poor oral hygiene, an exposed rough surface can lead to increased bacterial plaque accumulation and eventually peri-implantitis [11]. However, evidence for the influence of the implant surface characteristics as a risk indicator for peri-implantitis is very limited [41, 42]. In overall, there is controversy in the literature regarding the effect of surface characteristics and peri-implantitis. Based on two systematic reviews in 2008 and 2011, due to limited data available in the literature, there is no evidence that implant surface characteristics can have a significant influence on the initiation of peri-implantitis [41, 42]. Moreover, Persson et al. investigated the effect of surface roughness on the healing following peri-implantitis treatment in the beagle dog [43]. They found further amount of reosseointegration in implants with a rough surface, likely because the rough surface can facilitate the stability of the blood clot during the early phase of healing.

The advantages of rough-surfaced dental implants could be described by provided support for the coagulum development and facilitating greater bone healing and better qualified osseointegration. Perhaps hybrid implants should be considered in individuals that are highly predisposed to periodontitis [42]. In such cases, inflammation due to a rough or badly designed superstructure causes bone loss and exposed smooth threads. If in such patients the smooth treads become exposed in the oral cavity, the threads may be less plaque retentive, increasing the possibility of arresting the progression of peri-implantitis.

In the current study, crystallinity was measured according to the procedure described by Karamian et al. [44]. As observed in the *X*_*c*_ results, NHA 10% zircon has less crystallinity and more amorphous structure. The dissolution and precipitation behaviour of apatites are the principle factors governing their bioactivity [45]. The obtained SEM micrographs (Figure 5) show the formation and growth of apatite crystals on the surfaces of the NHA 10% zircon coating after soaking in the SBF solution during several periods of time. SEM observations showed amorphous and glassy surface structure, leading to more biodegradation of NHA 10% zircon coating compared to classic HA-coated implants. Dissolution of NHA/zircon coating may have significant advantages, such as an increase in calcium and phosphate. The presence of calcium is considered to regulate initiation of messenger ribonucleic acid (mRNA) transcription and protein synthesis and therefor lead to cellular differentiation [46]. It was reported that more soluble phases on the coatings might be more favourable for a stable interface with the biological environment [47]. It might be due to the release of calcium ions necessary for bone formation. As shown, the amount of calcium release in the NHA 10% zircon was three times more than that in classic HA coating (Figure 6(a)). On the other hand, delamination or biodegradation of the coatings might be partly responsible for the failure in the implant-coating interface [48, 49]. These contradictions need to be evaluated by further future studies.

Clinical data recommend that HA-coated implants may be valuable treatment indications when placing implantsin type IV bone, in fresh extraction sites, and in grafted maxillary or nasal sinuses, or when using shorter implants (less than or equal to 10 mm) [9]. Thus, prepared NHA/zircon coating compared to classic HA coating could be more useful in the aforementioned situations due to the higher bioactivity and roughness.

Zircon is a nonresorbable metal which provides good mechanical properties, superior to other ceramic materials, such as high bioactivity and roughness. Zircon seems to be suitable for dental application due to its good chemical and material stability, high strength, and proper roughness. However, considering all the limitations of current work, more studies are needed about the novel NHA/zircon coating. Besides, NHA/zircon as a new material needs extensive* in vivo* and long-term controlled studies.

## 5. Conclusion

The maximum *R*_*a*_ (14.54 *μ*m) was found in the sample containing 10% wt. ZrSiO_4_. In addition, the maximum calcium released (115 ppm) was found in this sample, which depends on two reasons: (a) increase of roughness and (b) decrease of crystallinity (to 41.1%). NHA/zircon nanocomposite coating possessed a good bioactivity and roughness and could be suitable for hard tissue formation in the field of biomedical implants. Further studies are needed to validate the results of this survey.

## Figures and Tables

**Table 1:** Chemical composition of NHA (XRF results).

Compound	Concentration (w/w %)
Ca	75.91
P	20.09
Na	1.25
F	1.20
Mg	0.708
Sr	0.151
Cl	0.200
Si	0.150
S	0.073
Al	0.061
Cu	0.056
Zn	0.053
Fe	0.052
K	0.038
Zr	0.008

LOI*	0.376

Total	100

*Loss on ignition (1000°C, 2 h).

**Table 2:** Nominal ion concentrations of SBF in comparison with those in human blood plasma.

Ion	Ion concentrations (m mol)	
	SBF	Human blood plasma
Na^+^	142.0	142.0
K^+^	5.0	5.0
Mg^2+^	1.5	1.5
Ca^2+^	2.5	2.5
Cl^−^	147.8	103.0
HCO_3_^−^	4.2	27.0
HPO_4_^2−^	1.0	1.0
SO_4_^2−^	0.5	0.5
pH	7.4	7.2–7.4

**Table 3:** Surfaces roughness results.

Experimental name	Composite (zircon %)	*R*_*a*_ (*μ*m)	Average *R*_*a*_ (*μ*m)
N1	0	12.92	12.63
N2	0	13.97	
N3	0	11	

N4	5	13.59	14.30
N5	5	15.93	
N6	5	13.39	

N7	10	15.55	14.54
N8	10	13.77	
N9	10	14.30	

N10	15	11.86	12.85
N11	15	13.44	
N12	15	13.24	

**Table 4:** The HA biodegradation properties of all the samples in the SBF.

Samples	Ca^2+^ release in SBF after 1 week (ppm)	Ca^2+^ release in SBF after 2 weeks (ppm)	WL. % of samples in SBF after 1 week (mg)	WL. % of samples in SBF after 2 weeks (mg)
NHA 0% zircon	36	78	1.13	0.28
NHA 5% zircon	40	90	2.40	2.80
NHA 10% zircon	62	115	3.31	4.02
NHA 15% zircon	48	96	0.25	1.11

**Table 5:** Crystallographic Parameters of the HA Phase of all the Samples.

Samples	Crystallinity (*X*_*c*_ %)	Crystallite Size ± 1 (nm)
NHA 0% Zircon	44.8	25.5
NHA 5% Zircon	43.2	28
NHA 10% Zircon	41.1	32
NHA 15% Zircon	42.9	28.2

## References

[1] Albrektsson T, Branemark PI, Hansson HA, Lindstrom J (1981). Osseointegrated titanium implants. Requirements for ensuring a long-lasting, direct bone-to-implant anchorage in man. *Acta Orthopaedica Scandinavica*.

[2] Dohan Ehrenfest DM, Coelho PG, Kang BS, Sul YT, Albrektsson T (2010). Classification of osseointegrated implant surfaces: materials, chemistry and topography. *Trends in Biotechnology*.

[3] Wennerberg A, Albrektsson T (2009). Effects of titanium surface topography on bone integration: a systematic review. *Clinical Oral Implants Research*.

[4] Rodrigues CVM, Serricella P, Linhares ABR (2003). Characterization of a bovine collagen-hydroxyapatite composite scaffold for bone tissue engineering. *Biomaterials*.

[5] Garcia R, Doremus RH (1992). Electron microscopy of the bone-hydroxylapatite interface from a human dental implant. *Journal of Materials Science: Materials in Medicine*.

[6] Harada Y (1989). Experimental studies of healing process on compound blocks of hydroxyapatite (HAP) particles and tricalcium phosphate (TCP) powder implantation in rabbit mandible—comparison of HAP/TCP ratios and plastic methods. *Shika Gakuho. Dental Science Reports*.

[7] Hench LL (1991). Bioceramics: from concept to clinic. *The American Ceramic Society Bulletin*.

[8] Webster TJ, Ergun C, Doremus RH, Bizios R (2002). Hydroxylapatite with substituted magnesium, zinc, cadmium, and yttrium. II. Mechanisms of osteoblast adhesion. *Journal of Biomedical Materials Research*.

[9] Biesbrock AR, Edgerton M (1995). Evaluation of the clinical predictability of hydroxyapatite-coated endosseous dental implants: a review of the literature. *The International journal of oral & maxillofacial implants*.

[10] Mouhyi J, Dohan Ehrenfest DM, Albrektsson T (2012). The peri-implantitis: implant surfaces, microstructure, and physicochemical aspects. *Clinical Implant Dentistry and Related Research*.

[11] Zetterqvist L, Feldman S, Rotter B (2010). A prospective, multicenter, randomized-controlled 5-year study of hybrid and fully etched implants for the incidence of peri-implantitis. *Journal of Periodontology*.

[12] Choi JW, Kong YM, Kim HE, Lee IS (1998). Reinforcement of hydroxyapatite bioceramic by addition of Ni3Al and Al2O3. *Journal of the American Ceramic Society*.

[13] Hench LL, Wilson J (1993). *An Introduction to Bioceramics*.

[14] Kim S, Kong YM, Lee IS, Kim HE (2002). Effect of calcinations of starting powder on mechanical properties of hydroxyapatite-alumina bioceramic composite. *Journal of Materials Science: Materials in Medicine*.

[15] Kong YM, Kim S, Kim HE, Lee IS (1999). Reinforcement of hydroxyapatite bioceramic by addition of ZrO2 coated with Al2O3. *Journal of the American Ceramic Society*.

[16] Lee IS, Whang CN, Kim HE, Park JC, Song JH, Kim SR (2002). Various Ca/P ratios of thin calcium phosphate films. *Materials Science and Engineering C*.

[17] Levingstone TJ (2008). *Optimisation of Plasma Sprayed Hydroxyapatite Coatings*.

[18] Wu C, Chang J, Ni S, Wang J (2006). In vitro bioactivity of akermanite ceramics. *Journal of Biomedical Materials Research A*.

[19] Sridhar TM, Kamachi Mudali U, Subbaiyan M (2003). Sintering atmosphere and temperature effects on hydroxyapatite coated type 316L stainless steel. *Corrosion Science*.

[20] Kokubo T, Takadama H (2006). How useful is SBF in predicting in vivo bone bioactivity?. *Biomaterials*.

[21] Jinawath S, Pongkao D, Yoshimura M (2002). Hydrothermal synthesis of hydroxyapatite from natural source. *Journal of Materials Science: Materials in Medicine*.

[22] Monshi A, Attar SS (2010). A new method to measure nano size crystals by scherrer equation using XRD. *Majlesi Journal of Materials Engineering*.

[23] Kokubo T, Ito S, Huang ZT (1990). Ca, P-rich layer formed on high-strength bioactive glass-ceramic A-W. *Journal of Biomedical Materials Research*.

[24] Kokubo T, Kushitani H, Ohtsuki C, Sakka S, Yamamuro T (1992). Chemical reaction of bioactive glass and glass-ceramics with a simulated body fluid. *Journal of Materials Science: Materials in Medicine*.

[25] Li P, Ohtsuki C, Kokubo T (1993). Effects of ions in aqueous media on hydroxyapatite induction by silica gel and its relevance to bioactivity of bioactive glasses and glass-ceramics. *Journal of Applied Biomaterials*.

[26] Posner AS (1985). The mineral of bone. *Clinical Orthopaedics and Related Research*.

[27] Soballe K, Brockstedt-Rasmussen H, Hansen ES, Bunger C (1992). Hydroxyapatite coating modifies implant membrane formation: controlled micromotion studied in dogs. *Acta Orthopaedica Scandinavica*.

[28] Soballe K, Hansen ES, Rasmussen BH, Jorgensen PH, Bunger C (1992). Tissue ingrowth into titanium and hydroxyapatite-coated implants during stable and unstable mechanical conditions. *Journal of Orthopaedic Research*.

[29] Rivero DP, Fox J, Skipor AK, Urban RM, Galante JO (1988). Calcium phosphate-coated porous titanium implants for enhanced skeletal fixation. *Journal of Biomedical Materials Research*.

[30] van Blitterswijk CA, Hesseling SC, Grote JJ, Koerten HK, de Groot K (1990). The biocompatibility of hydroxyapatite ceramic: a study of retrieved human middle ear implants. *Journal of Biomedical Materials Research*.

[31] Ichikawa Y, Akagawa Y, Nikai H, Tsuru H (1992). Tissue compatibility and stability of a new zirconia ceramic in vivo. *The Journal of Prosthetic Dentistry*.

[32] Albrektsson T, Hansson HA, Ivarsson B (1985). Interface analysis of titanium and zirconium bone implants. *Biomaterials*.

[33] Assal P (2012). The osseointegration of zirconia dental implants. *Schweizer Monatsschrift für Zahnmedizin*.

[34] Romanos GE, Johansson CB (2005). Immediate loading with complete implant-supported restorations in an edentulous heavy smoker: Histologic and histomorphometric analyses. *International Journal of Oral and Maxillofacial Implants*.

[35] Crespi R, Capparé P, Gherlone E, Romanos GE (2008). Immediate versus delayed loading of dental implants placed in fresh extraction sockets in the maxillary esthetic zone: a clinical comparative study. *International Journal of Oral and Maxillofacial Implants*.

[36] Borsari V, Giavaresi G, Fini M (2005). Physical characterization of different-roughness titanium surfaces, with and without hydroxyapatite coating, and their effect on human osteoblast-like cells. *Journal of Biomedical Materials Research B: Applied Biomaterials*.

[37] Lazzara RJ, Testori T, Trisi P, Porter SS, Weinstein RL (1999). A human histologic analysis of osseotite and machined surfaces using implants with 2 opposing surfaces. *International Journal of Periodontics and Restorative Dentistry*.

[38] Lang NP, Jepsen S (2009). Implant surfaces and design (Working Group 4). *Clinical Oral Implants Research*.

[39] Piattelli A, Cosci F, Scarano A, Trisi P (1995). Localized chronic suppurative bone infection as a sequel of peri-implantitis in a hydroxyapatite-coated dental implant. *Biomaterials*.

[40] Teughels W, Van Assche N, Sliepen I, Quirynen M (2006). Effect of material characteristics and/or surface topography on biofilm development. *Clinical Oral Implants Research*.

[41] Heitz-Mayfield LJA (2008). Peri-implant diseases: diagnosis and risk indicators. *Journal of Clinical Periodontology*.

[42] Renvert S, Polyzois I, Claffey N (2011). How do implant surface characteristics influence periimplant disease?. *Journal of Clinical Periodontology*.

[43] Persson LG, Berglundh T, Sennerby L, Lindhe J (2001). Re-osseointegration after treatment of peri-implantitis at different implant surfaces. *Clinical Oral Implants Research*.

[44] Karamian E, Khandan A, Eslami M, Gheisari H, Rafiaei N (2014). Investigation of HA vanocrystallite size crystallographic characterizations in NHA, BHA and HA pure powders and their influence on biodegradation of HA. *Advanced Materials Research*.

[45] Zhang Q, Chen J, Feng J, Cao Y, Deng C, Zhang X (2003). Dissolution and mineralization behaviors of HA coatings. *Biomaterials*.

[46] Gregoire M, Orly I, Menanteau J (1990). The influence of calcium phosphate biomaterials on human bone cell activities. An in vitro approach. *Journal of Biomedical Materials Research*.

[47] Van Blitterswijk C, Leenders H, van der Brink J, Bovell Y, Flach J, de Bruijn J (1993). Degradation and interface reactions of hydroxyapatite coatings: effect of crystallinity. *Transactions of the Society for Biomaterials*.

[48] Chang YL, Lew D, Park JB, Keller JC (1999). Biomechanical and morphometric analysis of hydroxyapatite-coated implants with varying crystallinity. *Journal of Oral and Maxillofacial Surgery*.

[49] Lee JJ, Rouhfar L, Beirne OR (2000). Survival of hydroxyapatite-coated implants: a meta-analytic review. *Journal of Oral and Maxillofacial Surgery*.

